# A Longitudinal Study of Hand Motor Recovery after Sub-Acute Stroke: A Study Combined fMRI with Diffusion Tensor Imaging

**DOI:** 10.1371/journal.pone.0064154

**Published:** 2013-05-28

**Authors:** Wenjuan Wei, Lijun Bai, Jun Wang, Ruwei Dai, Raymond Kai-yu Tong, Yumei Zhang, Zheng Song, Wen Jiang, Chuanying Shi, Mengyuan Li, Lin Ai, Jie Tian

**Affiliations:** 1 Key Laboratory of Molecular Imaging and Functional Imaging, Institute of Automation, Chinese Academy of Sciences, Beijing, China; 2 State Key Laboratory of Cognitive Neuroscience and Learning, Beijing Normal University, Beijing, China; 3 Department of Radiology, Beijing Tiantan Hospital, Capital Medical University, Beijing, China; 4 Department of Health Technology and Informatics, Hong Kong Polytechnic University, Beijing, China; 5 Department of Neurology, Beijing Tiantan Hospital, Capital Medical University, Beijing, China; National Yang-Ming University, Taiwan

## Abstract

Previous studies have shown that motor recovery of stroke can be assessed by the cortical activity and the structural integrity of the corticospinal tract (CST), but little is known about the relation between the cortical activity and the structural integrity during motor recovery. In the present study, we investigated the changes in brain activities evoked by twenty days’ functional electrical stimulation (FES) training in twelve sub-acute stroke patients with unilateral upper-limb disability. We compared cortex activity evoked by wrist movement of eleven stroke patients to that of eleven age-matched healthy subjects to figure out how cortex activity changed after stroke. We also measured the structural integrity represented by the fractional anisotropy (FA) asymmetry of the posterior limb of the internal capsule (PLIC) to find the relationship between the brain activity and the structure integrity. In our study, we found that patients with sub-acute stroke have shown greater activity in the contralesional primary motor cortex (M1) during the affected hand’s movement compared with healthy group, while the activity in ipsilesional M1 was decreased after the therapy compared to that before therapy, and the contralesional non-primary motor cortex showed greater activity after therapy. At the baseline we found that the positive correlation between the FA asymmetry of PLIC and the contralesional non-primary motor cortex activity showed that the greater damaged CST, the greater contralesional non-primary motor cortex recruited. While the negative correlation between them after the FES training indicates that after recovery the non-primary motor cortex plays different role in different stroke phases. Our study demonstrates that functional organization of a residual distributed motor system is related to the degree of disruption to the CST, and the non-primary motor areas plays an important role in motor recovery.

## Introduction

Stroke is a major cause of long-term disability in adults throughout the world, and the majority of post-stroke people endure motor impairment, especially a paretic hand. Recent studies on the recovery from stroke have demonstrated that the adult brain is capable of regeneration and compensation for motor deficits [1]. Given such plasticity of the brain, many rehabilitative procedures have been applied to improve the motor function in people with stroke [2].

Among these compensatory strategies, functional electrical stimulation (FES) is one of the effective and practical methods. This technology generally uses short durations of electrical pulses applied through the skin to drive paretic muscles activated, in order to improve patients’ grasping, walking and coordinated movements [3], [4], [5], [6]. FES has been demonstrated to be effective to accelerate the degree and rate of motor recovery. However, it is still unknown how the brain functionally and anatomically changes resulting from training of FES for the patient with motor deficits.

Functional magnetic resource imaging (fMRI) has been proved to be an important way to investigate motor recovery after stroke [1], [7], [8]. However, lots of previous fMRI studies of dynamic functional reorganizations of motor-related cortices after stroke are variable and the conclusions are controversial [1]. Some longitudinal studies suggest that neural activities in cortical motor areas are enhanced in both hemispheres after 2 weeks post-stroke and then decrease as a function of recovery in the primary and non-primary motor regions when performing movements of the affected limb [9], [10]. Other reports show that the motor recovery accompanies with the increased activation of the ipsilateral premotor cortex and the secondary somatosesory cortex. In other words, when the activity of the ipsilateral premotor cortex is enhanced by motor training, the greater such activation increase, the better is the recovery [11], [12].

For stroke patients with upper limb disability, an assessment of the corticospinal tract (CST) integrity could play an important role in rehabilitation studies. Many studies find that the structural integrity of fibers is correlated with motor impairments in stroke patients [13], [14], [15]. Diffusion tensor imaging (DTI) could provide information about the property of water diffusion, the extent of diffusion anisotropy and its orientation, which make it possible to be used to reconstruct three-dimensional images of white matter fiber tract [16]. Fractional anisotropy (FA), as an index of the diffusion characteristics of water molecules directed along the axis of major axonal pathway, can be used to measure the integrity of fiber tract. Reduced FA is interpreted as evidence of Wallerian Degeneration (WD) and axonal loss after stroke [17], [18]. And regional FA is used to predict motor impairments [19], [20], [21]. Functional MRI studies in stroke patients with affected hand movement have found that the brain activation areas include the primary motor cortex and non-primary motor cortex. The descending motor fibers originating from these areas all pass through the PLIC region. FA in the posterior limb of the internal capsule (PLIC) is a significant predictor of motor outcomes, compared to the FA of other regions, such as corona radiate, centrum semiovale [19] and cerebral peduncle [22]. And the FA in the PLIC has reliability and validity as well as the tract-specific in assessment of CST integrity [22].

Recently, some studies find that activity in the hemispheres is correlated with the CST integrity [14], [21], [23], [24]. Lotze et al. report that the contralesional hemisphere is often more pronounced in patients with greater CST damages [25], whereas Ward et al find that increased activity in the ipsilesional hemisphere is depended on the integrity of the CST, and impaired integrity of the CST is demonstrated to be associated with the primary motor cortex [23]. Although those studies have provided insights into the recovery mechanisms after stroke, little is known about the relationships of the cortex activity and the structural integrity during the brain reorganization.

The present study aims to figure out the relation between the cortex activities and the white matter integrity in stroke patients. First, we try to figure out which cortex activities changes after stroke by comparing the activity evoked by simple wrist movement in stroke patients with that of healthy subjects and how the cortex activities (mainly the primary and non-primary motor cortex activities) change after motor recovery in stroke patients. Second, we figure out the relationships of the cortex activity and the structural integrity during the rehabilitation of sub-cortical stroke patients considering FA of PLIC can predict the motor outcome of stroke patients.

## Materials and Methods

### Ethics Statement

Written informed consent was obtained from all subjects. The data was analyzed anonymously, and all research procedures were approved by the Beijing Tiantan Hospital Subcommittee on Human Studies and conducted in accordance with the Declaration of Helsinki.

### Subjects

Patients being diagnosed of ischemic stroke by MRI with unilateral upper-limb disability were recruited from the Beijing Tiantan Hospital. The criteria for patients recruited are listed as follows: (1) sub-acute stroke patients: >2 weeks and <6 weeks after the onset of stroke (first episode of stroke); (2) sufficient cognition to follow simple commands, MMSE (Mini-Mental State Examination score)>21; (3) no skin allergy to the FES stimulation electrodes; (4) Score “0” in finger mass extension sub-item of the Fugl-Meyer Assessments. Patients were excluded if they met any criteria below: (1) bilateral infarcts (2) recurrent stroke (3) any previous history of alcohol or drug abuse (4) history of epilepsy or other neurological disease and psychiatric disorder (5) serious cognitive deficits, comprehensive aphasia (6) other MRI contraindications (such as claustrophobia, etc). Finally, twelve patients met the criteria and participated in this study (8 males and 4 females, mean age = 52.75±12.5 year). Table 1 summarizes the demographic and information about those stroke patients. 11 age-matched and sexual-matched normal subjects (6 males and 5 females, mean age = 58.1±13.4 year) who were also recruited from Beijing Tiantan Hospital served as healthy controls. Each of them has normal neurological examination, no history of epilepsy or other neurological disease, psychiatric disorder and other MRI contraindications (such as claustrophobia, etc). All of the patients and the normal subjects are right-hand dominance.

**Table 1:** Demographic and stroke characteristics.

subject	sex	Age (years)	lesion	Lesion(mm^3^)	% of CSTaffected	MMSE	FM score		ARAT score		MAS score	
**1**	F	58	R BG	224	37.3	30	49	66	41	57	13	18
**2**	M	75	L CR	768	10.55	27	50	55	24	57	13	18
**3**	M	40	L BG CR	240	11.31	28	17	59	6	47	5	17
**4**	F	64	L PON	248	0	30	26	55	4	49	7	15
**5**	F	30	R BG	760	29.7	30	6	51	0	57	1	12
**6**	M	50	R brain-stem	88	0	29	44	62	57	57	14	17
**7**	M	53	R PON	176	0	30	44	63	47	57	15	17
**8**	M	58	R PON	432	0.16	30	4	41	0	40	1	12
**9**	F	63	R PON	224	0	29	34	66	49	57	11	18
**10**	M	42	L PLIC	280	22.13	26	√					
**11**	M	58	L BG	304	0	>21		√				
**12**	M	42	L PLIC	224	10.43	>21		√				

‘√’means that the data is only used for assess the improvement of motor function.

### Clinical Assessments

Each patient underwent a series of clinical evaluations. Clinical outcomes measurements included the upper extremity motor section of the Fugl-Meyer Asessment (FMA) scale, the responsiveness of the Action Research Arm (ARA) test, the upper limb Motor Assessment Scale (UL-MAS). The FMA is a system for assessing patients’ movement performance, including the hyperreflexia, flexion and extension synergies, and the ability to perform selective movements [26]. The maximum sum score of FMA is 66.The ARA test is used to measure arm function such as the ability to perform gross movements, grasp, move and release objects differing in size, weight and shape [27]. The maximum sum score of ARA is 57.The MAS have been shown to be a reliable measure of the upper limb function for adults following stroke [28]. It consists of ‘Upper Arm Function’, ‘Hand Movement’ and ‘Advanced Hand Activities’ items of MAS [29] and the maximum is 18. And the more scores, the better behavior patients can achieve.

### Training and fMRI Task

**Figure 1:**
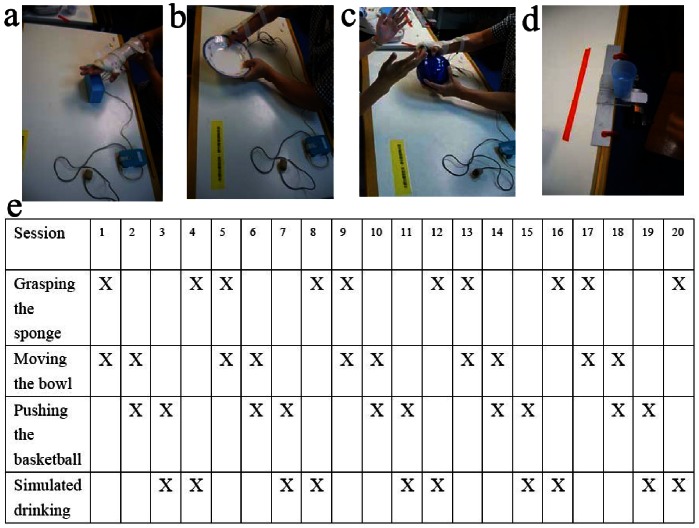
Bilateral training for therapy and the activities cycle in the 20-training sessions. **a**: grasping the sponge; **b**: moving the bowl; **c**: pushing the basketball; **d**: simulated drinking; **e**: the activities cycle in the 20-training sessions.

Patients were trained 5 days per week for 4 weeks. The rehabilitation therapy was bilateral training including grasping the sponge (See Figure 1a), moving the bowl (See Figure 1b), pushing the basketball (See Figure 1c) and simulated drinking (See Figure 1d). In each rehabilitation therapy, patients were asked to exercise two of the four bilateral training in random (See Figure 1e). Patients with FES therapy had 10 minutes passive movements, following by 20 minutes bilateral trainings with the FES stimulation and the stimulation electrodes were placed on the motor point of the extensor digitorium superficials and abductor pollicis longus muscles. At last the patients had 40 minutes traditional occupational therapy. Figure 1 shows the exact training for patients.

MRI scanning was taken before the 20 days’ therapy, as well as after the whole therapy for each patient (total two sessions). During fMRI scanning, a simple wrist movement was served as stimulation for patients. A simple block design was performed in which 20-second baseline and 20-second stimulation alternated and lasted for 4 minutes and 20 seconds, with 10 seconds rest beginning and ending in 10 seconds stimulation execution. And the healthy subjects had the same MRI procedure as the patients.

### Data Acquisition

Patients were scanned before and after the therapy. Functional images were acquired on a Siemens Trio 3 T scanner (Siemens, Erlangen, Germany) at Beijing Tiantan Hospital, and a custom-built head holder was used to prevent head movements. Thirty-one axial slices (thickness/gap = 3.5 mm/0.7 mm, matrix = 64×64, FOV = 200 mm×200 mm) parallel to the AC-PC plane and covering the whole brain were obtained using a T2*-weighted single-shot, gradient recalled echo planar imaging (EPI) sequence (TR = 2000 ms, TE = 30 ms, flip angle = 90°). High-resolution structural information on each subject was obtained using 3D MRI sequence with voxel size of 1 mm^3^ for anatomical localization (176 axial slices, TR = 1900 ms, TE = 2.13 ms, flip angle = 9°, FOV = 256 mm ×256 mm). DTI data were required using a single-shot spin-echo planar sequence, used an integrated parallel acquisition technique (iPAT) with an acceleration factor of 2 reduced the acquisition time and allowed the image with less distortion from susceptibility artifacts. Diffusion encoding scheme consisted of 30 directions with b = 1000 s/mm^2^ and 1 non-diffusion-weighted image (b0 = 0 s/mm^2^) (65 slices with 2 mm thickness, no gap; matrix = 124×128, TR = 11000 ms, TE = 94 ms, flip angle = 96.9°, FOV = 248 mm×256 mm).

### Lesion Maps

**Figure 2:**
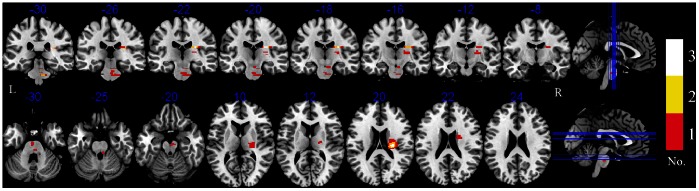
The lesion overlap of the patients. Lesion of each patient is drawn on each patient’s native space, and then normalized to the MNI space. The red means the number of lesions overlapped is 1, the yellow means 2 and the white means 3. L: the left hemisphere; R: the right hemisphere.

Prior to functional data analysis, we first calculated the percentage of the lesion affected in CST of each patient. Images from patients with left-side lesions were flipped at the midsagittal plane. Lesion masks were constructed from the DWI volume which is created in DTI data analysis showing the largest lesion extent using MRIcron (www.sph.sc.edu/comd/rorden/MRicron). Then we used the maximum probability map of the CST as defined in the SPM anatomy toolbox [30] to compute the intersection volume of the MNI normalized lesion, and the CST damage was expressed as the ration between the intersection volume and the total volume of the CST. There was no significant difference in DWI lesion volumes between the 2 sessions (paired t test, p = 0.062). The lesion overlap is shown in figure 2.

### fMRI Data Analysis

Functional images were analyzed using Matlab 7.6.0 (2008a) (Math works Inc., Natick, MA) and Statistical Parametric Mapping software (SPM 8, Wellcome Department of Imaging Neuroscience, London, UK, http://www.fil.ion.ac.uk). The first 10 time points of each task session were discarded due to the instability of the initial MRI signal and the subjects’ adaptation to the situation. For every subject, all the remaining volumes were realigned to the first image in order to correct for head motions, and co-registrated to the anatomical 3D image. As for the patients, all volumes were further spatially normalized to the Montreal Neurological Institute (MNI) space employing the masked lesion. The normalized images were further smoothed with a 6 mm full-width-at-half-maximum (FWHM) Gaussian kernel to compensate for residual variability after spatial normalization across subjects. As for the controls, the data processing is the same as the patients except the normalization, which all volumes that realigned and coregistrated were spatially normalized to the Montreal Neurological Institute (MNI) space.

Statistic analysis was performed in two steps. First, a single subject fixed effects model was used. The difference between thestimulation and the baseline was estimated at each voxel by using the general linear model (GLM) and the parameter for the covariate resulting from the least mean square fit of the model to the data were estimated, then the statistical parameter maps of the t statistic resulting from a linear contrast of the covariate were generated for each subject. The statistical threshold was set at P<0.05(corrected for multiple comparisons). In second-level analysis, the obtained individual t-maps were used in ‘random effect’ group analysis framework by one-sample t-test for different groups. In order to study the motor network reorganization, we investigated the longitudinal changes in task-related BOLD activities in stroke patients using the pair-t test. Further, we also performed the two-sample t-test to learn the changes of brain activity between the patients and the healthy subjects.

### DTI Data Analysis

DTI data were analyzed using Matlab 7.6.0 (2008a) and Explore DTI software [31]. Before analyzing the images, images from patients with lesion on the left side were flipped at the midsagittal plane so that all the lesions were on the same side [32]. For each subject, the diffusion-weighted images were registered to the b0 = 0 images and corrected for subject motion and geometric distortions induced by eddy current by incorporating the B-matrix rotation to preserve the orientational information correctly. Then the data were coregistrated to Montreal Neurological Institute (MNI) space. The diffusion tensor of each voxel was calculated by linear least-square method, and after diagonalization of the diffusion tensor, the diffusion tensor eigenvalues (λ_1_, λ_2_ and λ_3_), FA images and DW images were also calculated [33].

We then analyzed the hemisphere asymmetric by calculating the difference of FA between ipsilesional hemisphere and contralesional hemisphere. As we calculated the FA images and DWI images, we took each person’s lesion mask in the normalization, and then the data is used for VBM-style voxel-based statistics of FA images in SPM. Fractional anisotropy (FA) normalization was processed by FA-VBS in SPM 8 [34]. Firstly, we calculated a preliminary template using the affine transformed (FA) images. Secondly, images were registered to this preliminary template using nonlinear normalisation. Thirdly, we recalculated a further preliminary template using the normalised images until the influence of the template on the registration procedure was negligible (i.e. the difference between the generated templates were below 2%). The template generation procedure is part of the FA-VBS normalisation toolbox. Then the FA images were further smoothed with an 8 mm full-width-at-half-maximum (FWHM) Gaussian kernel. Statistic analysis was performed by paired-t test to detect the difference of FA in the two hemispheres, and the mask is thresholded at absolute 0.3 to reduce the gray matter and cerebro-spinal fluid influence, as well as to reduce the lesion effect.

To investigate the integrity of posterior limb of the internal capsule (PLIC), we calculated the FA of the contralesional and ipsilesional PLIC drawn based on existing anatomic knowledge about trajectories and anatomy [35], and FA asymmetry was calculated as
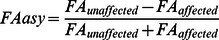
(1)


Where 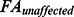
is the FA of contralesional PLIC, and 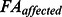
is the FA of ipsilesional PLIC, 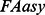
ranges from 0 (FA of PLIC in two hemispheres equals) to 1 (FA of PLIC in ipsilesional hemisphere is zero), and the greater of 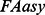
value, the more asymmetry of the hemispheres and the greater CST damage.

As we drew the ROIs manually, it was apt to be influenced by the rater’s familiarity with the brain anatomy and subjected to poor interrater reproducibility [22]. To establish the inter-rater reliabilities, the rater drew the ROIs twice with 3 weeks inter-analysis interval, and we calculated the interrater reliabilities for the mean FA of PLIC by interrater correlations (ICC) [36].

We also considered the changes of fractional anisotropy asymmetric in PLIC before and after therapy as an index to study the relationship of changes of structural integrity [24] and cortical activity. In the second-level analysis, we took 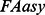, age and the FM score as regressors to find out which brain area activity was correlated with the changes of structural integrity partial out the variance of age factor and the clinical measurement factor before and after therapy.

## Results

### Clinical Assessments

**Figure 3:**
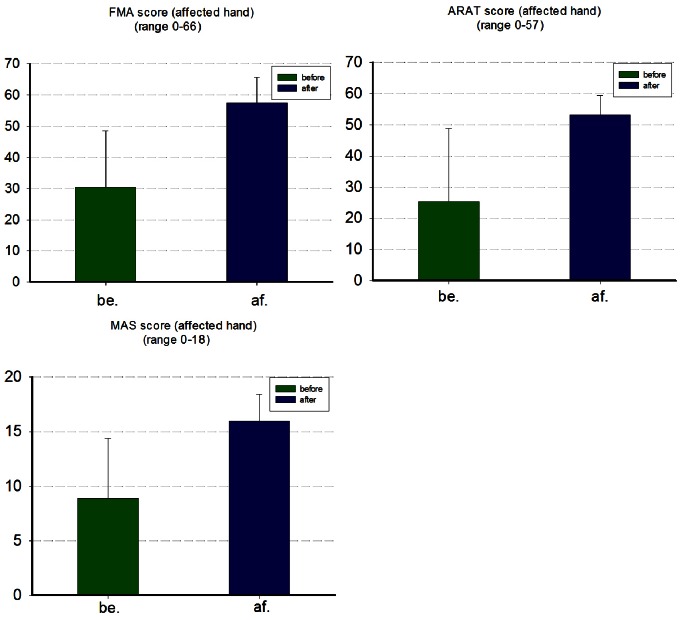
behavior data showing training clinical recovery. **A**: FMA score of the affected hand (p<0.001); **B**: ARAT score of the affected hand (p = 0.003); **C** MAS score of the affected hand (p<0.001). Error bars: standard error of the mean.

Patients with ischemia stroke in FES training had shown significant increases in hand movement by measuring their FMA (p<0.001), ARAT (p = 0.003) and MAS (p<0.001). As one patient had only taken part in the training before therapy, and the clinical assessments of the last two patients have missed, those data have only been analyzed to explore the brain activity during recovery. Figure 3 shows the changes of clinical assessment before and after the recovery therapy expressed as the mean score±SE. There were significant increases in the FMA (before: 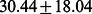; after: 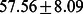), as well as the ARAT (before :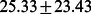; after: 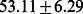) and MAS (before :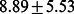; after: 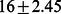).

The ICC for interrater reliability for the mean FA of PLIC in both the unaffected hemisphere (before therapy: 0.92; after therapy: 0.8) and affected hemisphere (before therapy: 0.99; after therapy: 0.98) showed that there was an excellent interrater reliability. We took the first measurement for further analysis.

### BOLD Signal Changes in Cortical Motor Network

**Figure 4:**
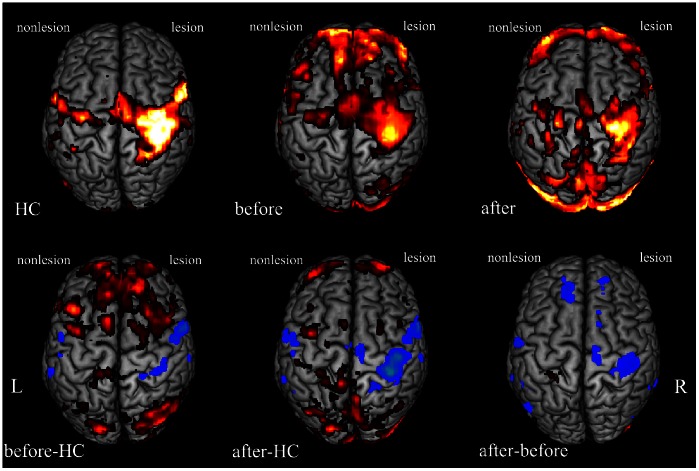
BOLD signal changes for affected hand movement at p<0.05 (corrected for multiple comparisons). First row: **HC**: brain activity for health controls; **before**: brain activity of patients before therapy; **after**: brain activity of patients after therapy; Second row: **before-HC**: brain activity of the difference between patients before therapy and healthy controls; **after-HC**: brain activity of the difference between patients after therapy and healthy controls; **after-before**: brain activity of the difference between patients before therapy and after therapy. Hot color indicates increased activity, and winter color indicates decreased activity. L: the left hemisphere; R: the right hemisphere.

Compared to the healthy subjects, patient group before therapy showed a decreased activity in bilateral ventrolateral premotor cortex (vPMC), ipsillesional M1 and ipsilesional somatosensory cortex, with an increased activity in contralesional M1, bilateral DLPFC, bilateral supplementary motor area (SMA), bilateral dPMC, bilateral posterior parietal cortex (PPC) at p<0.05(corrected for multiple comparisons) (see Table 2, see Figure 4d). Whereas compared to the activity of the healthy group, a decreased activity by the affected hand movement of the patient group after therapy was in the bilateral vPMC, ipsilesional dPMC, ipsilesional M1, ipsilesional DLPFC, ipsilesional somatosensory cortex and ipsilesional SMA, with an increased activity in bilateral dPMC, contralesional M1, contralesional SMA and bilateral PPC at p<0.05 (corrected for multiple comparisons) (see Table 3, see Figure 4e). Group results of the affected hand movement before therapy showed that movement of the affected hand activated the bilateralSMA, the bilateral M1, the contralesional vPMC, the bilateral dPMC, the ipsilesional PPC and the bilateral DLPFC at p<0.05(corrected for multiple comparisons) (see Figure 4b). After therapy, task-related activation was in the bilateral M1, bilateral SMA, contralesional PPC, contralesional vPMC, bilateral somatosensory cortex, bilateral DLPFC (see Figure 4c). Compared to task-related brain activity before therapy, patients with good recovery after therapy showed a decreased activity in the ipsilesional M1, the ipsilesional SMA, the contralesional somatosensory cortex and the contralesional vPMC (see Table 2, see Figure 4f).

**Table 2:** Cortex activity in stroke patients compared to healthy group before therapy.

regions	Side	Peak voxel				
		Talairach coordinate			t-value	Z-value
		x	y	z		
**Before>Health**						
**DLPFC**	L/contralesional	−44	27	37	3.605	3.06
	R/ipsilesional	14	58	34	3.286	2.85
**Somatosensory cortex**	L/contralesional	−18	−30	62	2.475	2.25
**Primary motor cortex**	L/contralesional	−18	−30	66	2.661	2.4
**dPMC**	R/ipsilesional	34	18	53	2.722	2.45
	L/contralesional	−40	9	55	3.329	2.88
**vPMC**	L/contralesional	−16	27	37	2.913	2.59
**SMA**	R/ipsilesional	16	15	62	2.5356	2.3
	L/contralesional	−10	19	60	3.716	3.14
**PPC (BA7)**	R/ipsilesional	44	−60	51	3.216	2.8
	L/contralesional	−16	−78	43	2.524	2.29
**Before<Health**						
**DLPFC**	R/ipsilesional	61	7	25	3.762	3.16
**Somatosensory cortex**	R/ipsilesional	34	−29	51	2.912	2.59
**Primary motor cortex**	R/ipsilesional	34	−27	51	3.245	2.83
**vPMC**	R/ipsilesional	61	5	26	3.485	2.98
	L/contralesional	−53	0	44	2.54	2.31

DLPFC: Dorsalateral Prefrontal cortex; dPMC: dorsolateral premotor cortex; SMA: Supplementary motor cortex;PPC: Posterior parietal cortex; vPMC: Ventrolateral premotor cortex.

**Table 3:** Cortex activity in stroke patients compared to healthy group after therapy.

regions	side	Peak voxel				
		Talairach coordinate			t-value	Z-value
		x	y	z		
**After>Health**						
**DLPFC**	R/ipsilesional	30	34	26	2.224	2.07
**Somatosensory cortex**	L/contralesional	−51	−19	54	2.38	2.2
**dPMC**	L/contralesional	−34	5	59	2.882	2.59
	R/ipsilesional	44	14	51	2.309	2.14
**SMA**	L/contralesional	−8	17	62	2.185	2.04
**PPC**	L/contralesional	−6	−63	53	3.312	2.91
	R/ipsilesional	4	−68	48	3.165	2.8
**After<Health**						
**DLPFC**	R/ipsilesional	61	9	24	3.889	3.29
**Somatosensory cortex**	R/ipsilesional	36	−30	57	4.556	3.7
**Primary motor cortex**	R/ipsilesional	36	−21	51	4.608	3.73
**dPMC**	R/ipsilesional	44	−14	60	4.175	3.47
**vPMC**	R/ipsilesional	59	6	37	2.911	2.61
	L/contralesional	−53	0	42	2.625	2.39
**SMA**	R/ipsilesional	12	−11	50	3.475	3.02
	L/contralesional	−10	−7	54	2.215	2.07
**After<Before**						
**DLPFC**	R/ipsilesional	20	52	34	2.166	2.01
**Somatosensory cortex**	R/ipsilesional	38	−28	57	2.441	2.23
**Primary motor cortex**	R/ipsilesional	36	−26	57	2.302	2.12
**vPMC**	L/contralesional	−61	−6	32	2.769	2.48
**dPMC**	R/ipsilesional	44	−16	60	2.248	2.07
**SMA**	R/ipsilesional	14	−16	67	1.983	1.85
**PPC(BA5)**	R/ipsilesional	28	−38	59	2.031	1.89

DLPFC: Dorsalateral Prefrontal cortex; dPMC: dorsolateral premotor cortex; SMA: Supplementary motor cortex; PPC: Posterior parietal cortex; vPMC: Ventrolateral premotor cortex.

**Figure 5:**
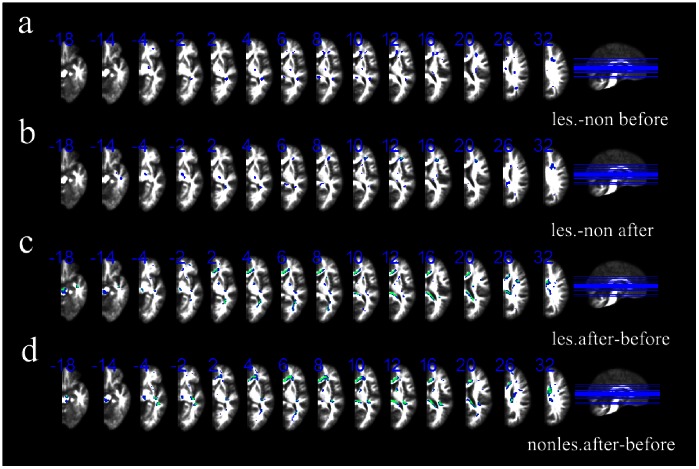
FA changes between the ipsilesional and contralesional hemisphere at p<0.05 (corrected for multiple comparisons). Figure 5a: the difference between the ipsilesional hemisphere and contralesional hemisphere before therapy; Figure 5b: the difference between the ipsilesional hemisphere and contralesional hemisphere after therapy; Figure 5c: the longitudinal changes of FA in the ipsilesional hemisphere; Figure 5d: the longitudinal changes of FA in the ipsilesional hemisphere. The winter color indicates the FA in the first hemisphere is smaller than the second one. L: the left hemisphere; R: the right hemisphere.

The longitudinal study of the voxel-wise FA showed that the FA of PLIC in the ipsilesional hemisphere after therapy was lower compared to that before therapy at p<0.05 (corrected for multiple comparisons), and the FA of cerebral peduncle in the ipsilesional hemisphere had the same change (see Figure 5c). The FA of PLIC in the contralesional hemisphere after therapy was lower compared to it before therapy, as well as the FA of cerebral peduncle in the contralesional hemisphere (see Figure 5d). The voxel-based analysis of FA images showed that the FA of PLIC in the ipsilesional hemisphere is lower than that in the contralesional hemisphere before therapy and after therapy at p<0.05 (corrected for multiple comparisons), whereas the FA of cerebral peduncle showed no difference between the two hemisphere no matter before therapy and after therapy. (see Figure 5a,5b).

### Regression Analysis

**Figure 6:**
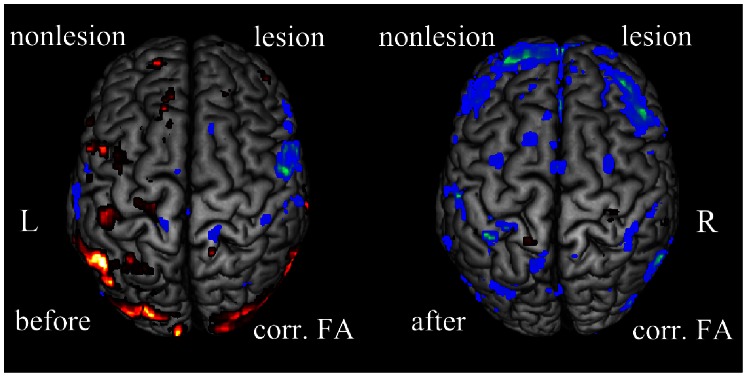
The correlation between the cortex activities and the FAasy at p<0.05 (corrected for multiple comparisons). Right: the correlation before therapy. Left: the correlation after therapy. The hot color indicates the positive correlation between the cortex activities and the FAasy; the winter color indicates the negative correlation between the cortex activities and the FAasy. L: the left hemisphere; R: the right hemisphere.

The results of regression analysis demonstrated a clear correlation between task-related brain activation within the motor system and the integrity of CST in sub-cortical stroke patients. Before therapy, the FA asymmetric in PLIC is positively correlated with contralesional M1, the contralesional dPMC, the contralesional vPMC, the contralesional somatosensory cortex, the contralesional DLPFC and the ipsilesional PPC, with a negative correlation in ispilesional vPMC, ipsilesional DLPFC and ipsilesional somatosensory cortex at p<0.05 (corrected for multiple comparsions) (see Figure 6a, see Table 4). Whereas the FA asymmetric in PLIC is negatively correlated with contralesional M1, contralesional SMA, bilateral dPMC, bilateral somatosensory cortex, bilateral DLPFC, bilateral PPC at p<0.05 (corrected for multiple comparsions) after therapy (see Figure 6b, see Table 5).

**Table 4:** Correlation between cortex activity and the FAasy in stroke patients before therapy.

regions	side	Peak voxel							
		Talairach coordinate				t-value		Z-value	
		x	y	z					
**Positive correlation**									
**DLPFC**	L/contralesional	−20	58	28		4.955		2.66	
**PPC**	R/ipsilesional	14	−45	41		5.965		2.88	
**Somatosensory cortex**	L/contralesional	−57	−16	30		5.134		2.71	
**Primary motor cortex**	L/contralesional	−20	−23	53		4.176		2.46	
**dPMC**	L/contralesional	−51	12	44		5.718		2.83	
**vPMC**	L/contralesional	−53	1	15		4.357		2.51	
**Negative correlation**									
**DLPFC**	R/ipsilesional	63	7		25		7.197		3.09
**Somatosensory cortex**	R/ipsilesional	65	−22		36		4.36		2.51
**vPMC**	R/ipsilesional	55	0		46		8.641		3.29
	R/ipsilesional	63	0		35		10.527		3.5

DLPFC: Dorsalateral Prefrontal cortex; dPMC: dorsolateral premotor cortex; SMA: Supplementary motor cortex; PPC: Posterior parietal cortex; vPMC: Ventrolateral premotor cortex.

**Table 5:** Correlation between cortex activity and the FAasy in stroke patients after therapy.

regions	side	Peak voxel				
		Talairach coordinate			t-value	Z-value
		x	y	z		
**Negative correlation**						
**DLPFC**	R/ipsilesional	4	33	30	6.911	3.05
	L/contralesional	−16	38	24	7.042	3.07
**PPC**	L/contralesional	−4	−68	40	3.453	2.23
	R/ipsilesional	2	−70	37	3.44	2.21
**Somatosensory cortex**	L/contralesional	−55	−19	38	11.47	3.59
**Primary motor cortex**	L/contralesional	−57	−18	36	7.717	3.17
**dPMC**	R/ipsilesional	32	7	60	2.726	1.94
	L/contralesional	−34	5	59	3.852	2.36
**SMA**	L/contralesional	−18	19	60	2.788	1.96

DLPFC: Dorsalateral Prefrontal cortex; dPMC: dorsolateral premotor cortex; SMA: Supplementary motor cortex; PPC: Posterior parietal cortex; vPMC: Ventrolateral premotor cortex.

## Discussion

In this longitudinal study we investigated the correlation between the brain activity and the CST integrity in stroke patients during recovery. Our study has also shown that movement of the affected hand produced a more bilateral motor network including primary and non-primary motor cortex compared to the healthy controls. Activity before therapy showed an overactivation in the primary and non-primary motor areas [9], such as the contralesional dPMC [37], ipsilesional dPMC, bilateral SMA, bilateral DLPFC, bilateral PPC. After therapy, the tasked-related brain activity in patients was clearly less recruited in ipsilesional hemisphere compared to the brain activity before therapy, demonstrating that stroke patients showed a reorganization during recovery [1]. Our study demonstrated that patients shown a changed activation of the motor network after recovery, suggesting that patients underwent brain reorganization during recovering, and the FA voxel-based analysis demonstrated that the patients experienced Wallerian Degeneration of the pyramidal tract.

### Brain Activity in Primary Motor Cortex

In our study, a decreased activation in ipsilesional M1 compared to the healthy group indicated that the ipsilesional M1 played an important role in hand movement of stroke patients, as fibers in the corticospinal originate mainly from the primary motor cortex. Many studies have also demonstrated that inhibition of the ipsilesional primary motor cortex induced poor motor function of the paretic hand in subcortical stroke patients by ways of transcranial magnetic stimulation (TMS) [38], [39]. We also found a decreased activity in ipsilesional M1 in the patients after recovery compared to healthy group and the patients before therapy, and therefore suggested that the improvement of motor performance in stroke patients may be induced by the contralesional M1 and other motor-related brain areas after the FES training.

Although the ipsilesional M1 plays the main role in motor execution in sub-cortical patients, the contraleisonal hemisphere could play a contributing role when the reorganization activity in the affected hemisphere is not enough to compensate for the motor deficits. Many studies about connectivity have demonstrated that the contralesional M1 had an effect on the ipsilesional M1 [40], [41], [42], but the role of the contralesional M1 in motor recovery after stroke remains controversial. In our study, before therapy, the FAasy positively correlated with brain activity in contralesional M1, indicating that the more damage of CST integrity, the more recruited of contralesional M1. Those findings support the idea that the contralesional M1 has a more compensatory role in motor execution in more damaged stroke patients before therapy. Our study is in line with Rehme et al study, which indicates that the increase in the activity of contralesional M1 correlates with the amount of spontaneous motor improvement in initially more impaired patient in early phase [7]. After recovery training, brain activity in contralesional M1 is increased compared to the healthy group, and it is negative correlated with FAasy, implicating that contralesional primary motor cortex is more activated in poorer motor performance patients during motor recovery after therapy. This result indicates that the contralesional M1 has different roles in motor recovery in different stroke phase. And some studies implicate that disrupting of contralesional M1 by repetitive TMS may lead to improvement of motor performance of the affected hand after 3 month or 12 month stroke [37], [43].

### Brain Activity in Secondary Motor Cortex

The substrates underlying motor recovery after stroke is not completely understood, some investigations have shed lighted on the non-primary motor areas studying stroke patients [11], [23], [44]. As the vPMC, the dPMC and the M1 all send outputs to the spinal cord, and the non-primary motor cortex provide additional capacity for the fast execution of movement, and such capacity may play a role in motor learning and in recovery of motor deficits [45]. Some studies examines the structural and functional relationships of the premotor areas and the spinal cord [45], [46], [47] and supports the idea that the increased activity of secondary motor areas, especially the bilateral dPMC, may be a compensating activation for CST damage [21], [23], [48].

In the present study, we can find an increased activity in bilateral dPMC compared to healthy group. The positive correlation between the FA asymmetry of PLIC and the activity of contralesional dPMC suggested that the greater damage of the CST integrity, the more recruitment of contralesional dPMC, indicating that the contralesional dPMC may play a compensate role in motor execution when moving the affected hand. After recovery, there was a significant negative correlation between the FA asymmetry of PLIC and the activity of ipsilesional dPMC, it means that after therapy the brain reorganization recruited more contralesional dPMC to executive the paretic hand movement, which indirectly support the idea that the recovery have more recruitment of the contralesional dPMC.

As dPMC and vPMC are involved in different motor network [49], these two areas have different roles played in motor recovery after stroke. The vPMC can be explained by a cardinal concept of direct sensorimotor processing, in other words, vPMC receives information on a motor target and sending outputs to achieve an action directly [50]. In our study, we observed a decreased activation in ipsilesional vPMC in stroke patients compared to the healthy controls. This finding suggested that the vPMC was less involved in hand movement for the stoke patients than for the healthy participants, which is opposite to the activity of dPMC. We also found a positive correlation between the contralesional vPMC and FAasy and negative correlation between the ipsilesional vPMC and FAasy before therapy suggested that patients with greater integrity of CST may have more ipsilesional vPMC and less contraleisonal vPMC recruited during motor execution,impling that the contralesional vPMC had a positive role in motor recovery after stroke and the inter-hemisphere competition.

### Brain Activity in Somatosensory Cortex

In our study, we also found significant changes in the superior parietal lobe (SPL) and S1 activity compared to the healthy subjects. Somatosensory input in the form of peripheral nerve stimulation can influence functional measures of motor performance, which could contribute to improved motor function after stroke [51]. The SPL is known to integrate sensory information and has been shown to be involved in sensorimotor integration for hand and arm coordination [52]. In our study, there was an increased activity in contralesional SPL compared to the health controls. We can also find a positive correlation between the brain activity in contralesional SPL and the FAasy, which means that the more damaged CST integrity, the more recruited of the contralesional SPL. Our findings are consistent with interhemispheric competition models of sensorimotor processing during motor recovery [53] and support the idea that the contralesional sensorimotor processing plays an important role in motor execution in stroke patients during motor recovery.

### Conclusions

Many treatments are being adopted to motor deficits in stroke patients [7], [25], most of which rely on promoting activity driven changes within surviving motor networks. Our results support the idea that functional organizations of the residual distributed motor system is related to the degree of disruption to the CST, and the contralesional hemisphere have an compensate role in motor execution in stroke patients, as well as the ipsilesional non-primary motor areas. We have not studied enough patients, whose onset of stroke varies and the lesions occurred in different hemispheres, to be able to draw firm conclusions, however, within our patient group, we have observed some remarkably relations between the functional organization of a residual distributed motor system and the degree of the CST integrity. In the future, we will combine the structure connectivity, for example, the fiber numbers between the brain areas of motor network as connection weight, with functional connectivity to get a more clear understanding of the roles of the primary and non-primary motor cortex in stroke reorganization.
